# Supplementary material for: Predictive and Prognostic Value of Serum Neutrophil Gelatinase-Associated Lipocalin for Contrast-Induced Acute Kidney Injury and Long-Term Clinical Outcomes after Percutaneous Coronary Intervention
Source: J Clin Med. 2022 Oct 10;11(19):5971. doi: 10.3390/jcm11195971 (PMC9573626; doi:10.3390/jcm11195971)
Supplement: Supplementary file 1 [file jcm-11-05971-s001.zip › jcm-1948807-supplementary.pdf]

Supplementary Table S1. Baseline clinical and angiographic characteristics

Variables	CI-AKI (-) (n=535)	CI-AKI (+) (n=98)	*P* value
Age (years)	63.0 ± 11.7	68.3 ± 11.8	0.011
Male	359 (67.5%)	56 (57.1%)	0.050
Body mass index (Kg/m^2)	24.9 ± 3.4	24.1 ± 3.8	0.028
Hypertension	379 (71.2%)	74 (75.5%)	0.463
Diabetes mellitus	196 (36.8%)	49 (50.0%)	0.018
Dyslipidemia	195 (36.7%)	24 (24.5%)	0.021
Current smoking	149 (28.0%)	30 (30.6%)	0.626
Family history of coronary artery disease	46 (8.6%)	7 (7.1%)	0.843
Prior stroke	53 (10.0%)	12 (12.2%)	0.473
Prior myocardial infarction	45 (8.5%)	7 (7.1%)	0.842
Prior percutaneous coronary intervention	74 (13.9%)	8 (8.2%)	0.142
Prior statin use	169 (31.8%)	22 (22.4%)	0.073
Clinical presentation			0.082
Stable angina pectoris	153 (28.8%)	20 (20.4%)	
Unstable angina pectoris	126 (23.7%)	18 (18.4%)	
NSTEMI	154 (28.9%)	41 (41.8%)	
STEMI	88 (16.5%)	17 (17.3%)	
Silent myocardial ischemia	11 (2.1%)	2 (2.0%)	
Ejection fraction (%)	55.8 ± 11.5	49.3 ± 14.6	<0.001
NGAL (ng/mL)	138.0±98.6	149.6±88.8	0.0279
Total cholesterol (mg/dl)	135.6 ± 33.1	130.0 ± 29.4	0.246
Triglyceride (mg/dl)	156.4 ± 190.4	151.0 ± 95.3	0.842
HDL cholesterol (mg/dl)	45.2 ± 10.9	41.6 ± 10.1	0.030
LDL cholesterol (mg/dl)	73.2 ± 23.4	70.7 ± 20.4	0.487
High-sensitivity C-reactive protein (mg/l)	9.5 ± 27.3	13.5 ± 31.6	0.195
eGFR (mL/min/1.73m^2)	71.5 ± 26.9	73.0 ± 34.2	0.685

eGFR <60 mL/min/1.73m^2	186 (34.8%)	39 (39.8%)	0.359
Hemoglobin (mg/dl)	13.5 ± 2.5	13.1 ± 2.3	0.107
Medications at discharge			
Aspirin	517 (97.2%)	96 (98.0%)	0.999
Clopidogrel	340 (63.9%)	54 (55.1%)	0.112
Potent P2Y12 inhibitor	191 (35.9%)	44 (44.9%)	0.111
Statins	527 (99.1%)	96 (98.0%)	0.299
Beta-blocker	368 (69.2%)	74 (75.5%)	0.231
Renin angiotensin system inhibitor	284 (53.4%)	59 (60.2%)	0.226
Hypotension	55 (10.3%)	9 (9.2%)	0.856
IABP or ECMO	2 (0.4%)	1 (1.0%)	0.398
Culprit coronary lesion			0.068
Left anterior descending	248 (47.6%)	58 (59.8%)	
Left circumflex	99 (19.0%)	21 (21.6%)	
Right	147 (28.2%)	16 (16.5%)	
Left main	26 (5.0%)	2 (2.1%)	
Multivessel	168 (31.6%)	36 (36.7%)	0.348
Contrast volume (ml)	217.9 ± 121.0	220.8 ± 115.6	0.831
Number of total stents	1.70 ± 1.03	1.82 ± 1.11	0.305
Mean diameter of stents (mm)	3.11 ± 0.43	3.08 ± 0.38	0.473
Total length of stents (mm)	43.6 ± 30.4	48.3 ± 36.6	0.175

Note: Values are number (%) or mean ± standard deviation

Abbreviation: NSTEMI, non-ST-segment elevation myocardial infarction; STEMI, ST-segment elevation myocardial infarction; HDL, high-density lipoprotein; LDL, low-density lipoprotein; eGFR, estimated glomerular filtration rate; IABP, intra-aortic balloon pump; ECMO, extracorporeal membrane oxygenation.
